# Metabolic transitions along a moisture gradient in a poly-extreme high-altitude desert ecosystem within the Atacama Desert

**DOI:** 10.1186/s40793-025-00847-7

**Published:** 2026-01-23

**Authors:** Diego Medina Caro, Alexander Bartholomäus, Ayón García, Rómulo Oses, Susanne Liebner, Dirk Wagner

**Affiliations:** 1 GFZ Helmholtz Centre for Geosciences, Section Geomicrobiology, Telegrafenberg, 14473 Potsdam, Germany; 2 Laboratorio de Investigación de la Criósfera de los Andes (LICA), Instituto de investigaciones científicas y tecnológicas (IDICTEC), Universidad de Atacama, 1530000 Copiapó, Chile; 3 Centro Regional de Investigación y Desarrollo Sustentable de Atacama (CRIDESAT), Universidad de Atacama, 1530000 Copiapó, Chile; 4 Institute of Biochemistry and Biology, University of Potsdam, 14476 Potsdam, Germany; 5 Institute of Geosciences, University of Potsdam, 14476 Potsdam, Germany

**Keywords:** Extreme environment, High-altitude desert, Metatranscriptomic, Energy metabolism, Community assembly

## Abstract

**Background:**

The Barrancas Blancas (BB) plain, situated in the high-altitude Atacama region, is a cryogenic, hyper-arid, poly-extreme environment. The seasonal formation of a temporary freshwater lake under these harsh conditions provides a unique opportunity to examine how liquid water influences the metabolic responses of desert microbial communities. Using a metatranscriptomic approach, we characterised microbial community responses along a 70-meter natural moisture gradient.

**Results:**

Along the gradient, the most active sites (8–23 m from the lake) exhibited high RNA recovery and diverse metabolic functions. Bacillariophyta (diatoms) drove oxygenic phototrophy, while Pseudomonadota contributed to anoxygenic phototrophy and nitrogen fixation. Additionally, Pseudomonadota, Actinomycetota, and Bacteroidota expressed genes for the oxidation of nitrate, sulfide, thiosulfate, and trace gases (H₂, CO). The energy derived from these processes was reflected in the high capacity for carbon fixation by these taxa. Moreover, network analysis revealed that these primary producers co-occurred with a diverse range of heterotrophic prokaryotic and eukaryotic groups. In contrast, at the driest site, hydrogen oxidation was the primary energy-conserving process, predominantly associated with Actinomycetota, which also contributed to hydrogenotrophic carbon fixation. Notably, even at this site, heterotrophic eukaryotes co-occurred with these chemolithotrophic primary producers.

**Conclusions:**

This study presents the first transcriptomic analysis from the high-altitude Atacama Desert, facilitated by the favourable moisture conditions. Furthermore, these findings highlight a moisture-driven transition in microbial energy acquisition strategies and emphasise the ecological significance of both photoautotrophy and chemolithotrophy, which likely vary depending on the dynamics of temporary lakes. The BB plain and its lake thus offer a robust model for understanding microbial resilience, functional plasticity, community assembly, and trophic interactions in extreme environments, providing novel insights into life at the edge of habitability.

**Graphical abstract:**

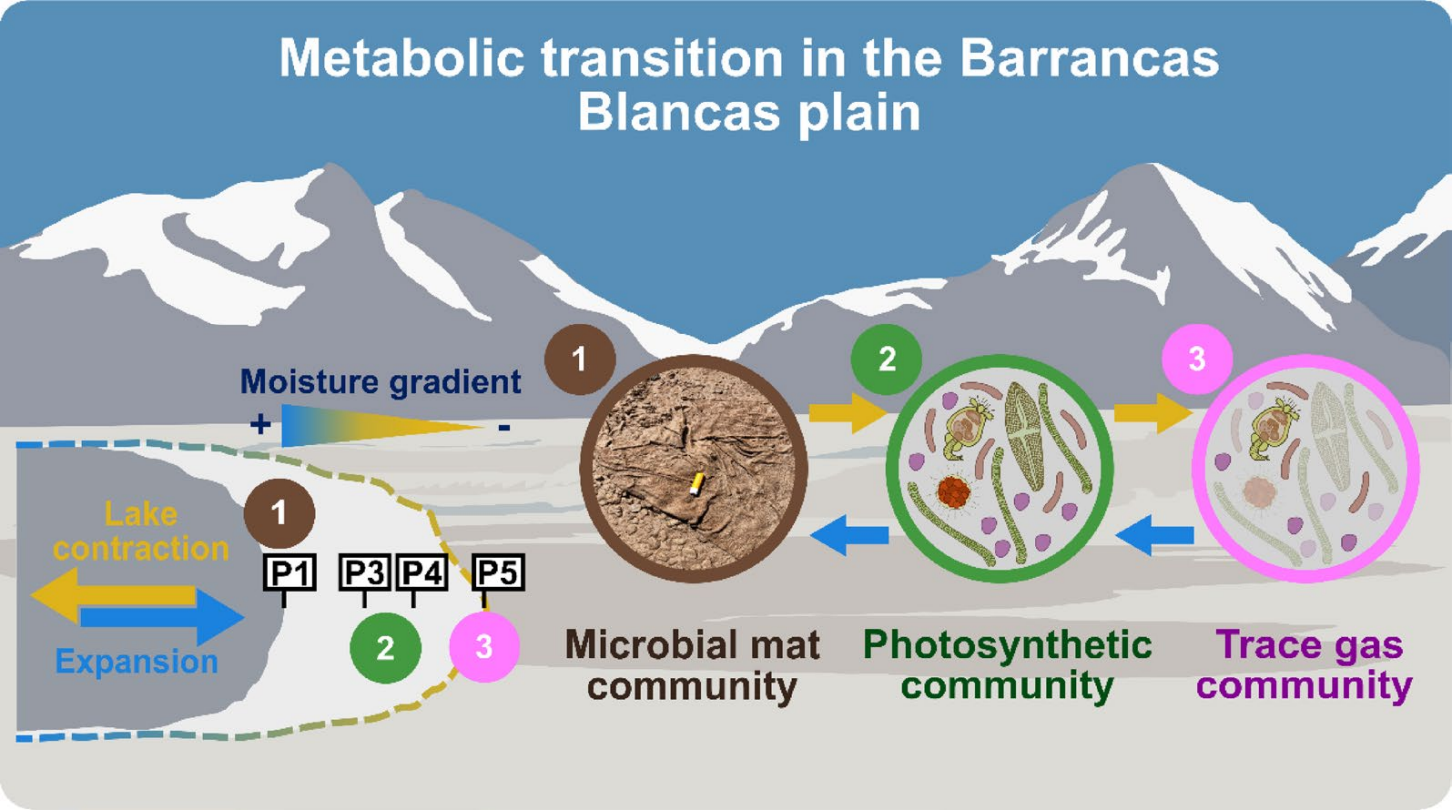

**Supplementary Information:**

The online version contains supplementary material available at 10.1186/s40793-025-00847-7.

## Background

 The Puna de Atacama is a high-altitude environment within the Atacama Desert [1]. This region is marked by extreme environmental conditions that lead to a negative water balance, mainly due to low water input, intense UV radiation, and high sublimation rates [2–6]. Water scarcity is the primary factor limiting life in these high-altitude environments, as it leads to low nutrient availability and biomass production, creating the oligotrophic conditions that define the region [7–9].

For many years, deserts were regarded as barren environments [10–13]. Nevertheless, in recent decades, scientific endeavours—particularly those utilising cultivation-independent methodologies—have disclosed that these extreme habitats are inhabited by highly diverse microbial communities (e.g [5, 9, 14–20]. , . The high microbial diversity present in such environments has conventionally been ascribed to the existence of phototrophic primary producers, which synthesise organic matter that supports the growth of heterotrophic community members [21, 22]. However, oxygenic phototrophs, including Cyanobacteriota and microalgae, are generally restricted to specific niches within the topsoil, such as biological soil crusts (biocrusts), or to lithic refugia to endure the poly-extreme conditions [22, 23]. Given that oxygenic phototrophs necessitate liquid water for photosynthesis, their activity is restricted to brief intervals following water influxes, such as morning dew or infrequent rainfall events [23–25].

In high-altitude volcanic soils, oxygenic phototrophic bacteria are typically absent when water is not present [9, 17, 26, 27]. Instead, these environments are predominantly characterised by chemoheterotrophic phyla, including Actinomycetota, Pseudomonadota, and Bacteroidota [14, 15, 17, 27–30]. Alternative primary production strategies, such as anoxygenic photosynthesis, which does not produce oxygen, have been frequently documented and recognised for their crucial role in the carbon cycle and primary production within Andean ecosystems because this process does not require water [9, 15, 30–35]. Anoxygenic phototrophs are typically associated with purple sulfur and non-sulfur bacteria belonging to the classes Alphaproteobacteria and Gammaproteobacteria [9, 33, 36] or are found within Chloroflexota taxa [37].

Furthermore, in recent years, there has been an increasing emphasis on the utilisation of inorganic compounds as energy sources, particularly concerning chemolithotrophic taxa that possess the capability to harness atmospheric trace gases such as H₂, CO, and CH₄ [22, 38–42]. Numerous bacteria prevalent in desert environments, notably members of the phylum Actinomycetota, have demonstrated the ability to oxidise H₂ to satisfy their fundamental metabolic requirements during periods of starvation or in conditions of limited water availability [40–45]. Some microorganisms even use the energy derived from this process for carbon fixation [40, 41, 46, 47]. Consequently, the capacity to metabolise trace gases has been proposed as a pivotal factor elucidating the resilience of highly diverse yet seemingly simplistic microbial communities exhibiting low-energy flux within poly-extreme desert settings [27, 40, 42, 47]. Nonetheless, the majority of investigations concerning trace gas metabolism have primarily concentrated on low-altitude deserts. In contrast, in high-altitude deserts, only studies employing DNA-based methodologies have indicated the potential significance of trace gas metabolism in these nutrient-scarce environments [9, 17, 27, 28, 48–53]. To this day, transcriptomic profiling of these processes remains absent, underscoring a significant gap in our comprehension of microbial strategies within extremely high-altitude locations.

The study site at the Barrancas Blancas (BB) plain, situated at an altitude of 4,500 to 5,000 m above sea level, represents a high-altitude cryospheric desert ecosystem within the Puna de Atacama, characterised by poly-extreme conditions. The uniqueness of this location is attributed to the presence of liquid water during summer, when a temporary freshwater lake forms, thereby creating a natural moisture gradient [54]. Our previous study indicated that the favourable conditions imposed by the lake sustained an active soil heterotrophic bacterial community along a 70-meter moisture gradient, with an increasing trend of activity occurring 8 to 23 m from the lake [54]. Moreover, oxygenic phototrophs, including Cyanobacteriota, were consistently identified in low abundance throughout the moisture transect [54], suggesting that anoxygenic phototrophic bacteria, chemolithoautotrophic bacteria, or even oxygenic phototrophic eukaryotes [55] could play a predominant role in soil energy metabolism. Furthermore, a shift between photoautotrophic and trace gas metabolisms has been hypothesised to occur in response to hydration levels [22, 56], rendering the moisture gradient at our study site an exemplary natural system for investigating this transition. To evaluate these hypotheses, we utilised a metatranscriptomic approach to characterise the metabolic strategies of both prokaryotic and eukaryotic communities along the moisture gradient and to investigate potential trophic interactions among community members further.

## Methodology

### Study site description and sample collection

The Barrancas Blancas (BB) plain is a high-altitude desert environment (4,500–5,000 m a.s.l.) located in the Puna de Atacama, Chile (27°02’S, 68°39’W; Figure S1a). It features a temporary lake, which expands and contracts throughout the season. Due to the unique presence of liquid water at this location, we conducted a moisture transect sampling to assess its impact on the living microbial community. Briefly, the transects (T1, T2, and T3) extended from the lake sediment (LS) and the shoreline of the temporary lake (soil pit 1; P1), passing through soil pits P2, P3, and P4, to a reference site with distinct morphological characteristics and reduced hydrological influence, located approximately 70 m away (P5; Figure S1b) [54].

During the BB campaign in March 2022, we collected samples from all soil pits down to a total depth of 30 cm. Depth layers were coded as follows: surface (0–5 cm) = “−1” (e.g., P3-1), 5–10 cm = “−2”, 10–20 cm = “−3”, and 20–30 cm = “−4”, following the methodology established in our previous study [54]. Additionally, in the same campaign, we collected samples for RNA-based analysis under sterile conditions, with all tools disinfected using filtered 70% ethanol. Samples were immediately stored in liquid nitrogen within a dry shipper (MVE Vapor Shipper Serie, Cryo-Moover) for transport from Chile to Germany. The dry shipper was continuously charged with liquid nitrogen until shipment, and the samples were kept in it for eight days during transit. Upon arrival in Germany, all samples were stored at 80 °C until further processing.

 For this study, we excluded the lake sediment (LS), soil pit 2 (P2), and the 5–10 cm and 10–20 cm depth layers. Instead, we focused on the most active samples—P3-1 and P4-1—identified in our previous work [54], and compared them with samples from the start (P1) and end (P5) of the transect, as well as with subsurface samples. In total, this selection resulted in 24 samples, which were analysed in this study (Table S1).

### iDNA extraction, RNA extraction, and library preparation for metagenomic and metatranscriptomic sequencing

For the metagenomic analysis, we utilised intracellular DNA (iDNA) extracts reported in Medina Caro et al. [54] and extracted as described in Medina Caro et al. [57]. Briefly, 3 g of soil were mixed with ice-cold sodium phosphate buffer (120 mM, pH 8.0), agitated, and centrifuged multiple times to collect a supernatant containing both iDNA and extracellular DNA (eDNA). The iDNA was separated from eDNA by filtration through a 0.22 μm Sterivex™ unit. Filters containing intact cells were then lysed using CTAB buffer, SDS, and phenol: chloroform: isoamyl alcohol. The DNA was purified with silica-based spin columns (Zymo Research, USA) and eluted in 100 µL of molecular-grade water. DNA concentrations were measured using the Qubit™ dsDNA High Sensitivity (HS) assay (Invitrogen; Waltham, United States).

For RNA extractions, we used 0.25 g of soil with the ZymoBIOMICS™ DNA/RNA Miniprep Kit. Additionally, we performed an in-column DNase digestion step to eliminate residual DNA contamination from RNA samples before further quantification, following the manufacturer’s instructions (Zymo Research; Irvine, United States). RNA samples were eluted in 50 µL of molecular-grade water. To meet the RNA content requirements for sequencing, samples were extracted multiple times (except P3-1 and P4-1), pooled and concentrated using the Centrifuge Concentrator Plus system (Eppendorf) in 15-minute intervals at room temperature, followed by 10 min on ice. RNA quality was assessed using the 4150 TapeStation System (Agilent Technologies; Santa Clara, United States) with the Agilent High Sensitivity RNA ScreenTape Assay (Agilent Technologies; Santa Clara, United States).

For metagenomic and metatranscriptomic sequencing, 100 ng of DNA and 20 ng of RNA per sample were shipped on dry ice to Eurofins Genomics (Ebersberg, Germany). Metagenomic and metatranscriptomic libraries were prepared using standard workflows with unique dual-indexing (UDI) for DNA and a strand-specific cDNA workflow for RNA, including mRNA fragmentation, random-primed cDNA synthesis, adapter ligation, and PCR amplification. Sequencing was performed on an Illumina NovaSeq6000 platform in paired-end mode (2 × 150 bp), targeting 10 million read pairs for DNA and 10 million reads for RNA.

### Metagenome analysis

Metagenomic data were processed using the ATLAS pipeline v2.12.0 [58], which includes an extended workflow for quality control, contig assembly, gene prediction and functional annotation. Among the included tools are several standard tools, e.g., metaSPAdes v3.15.3 for read assembly [59], eggNOG mapper v2.1 and eggNOG database v5.0 for functional gene annotation [60]. Default parameters were used, except for RAM (up to 1.5 TB) and CPU/threads (up to 80 threads). Gene taxonomy assignment to specific phyla was done using the phylum of the closest hit given by eggNOG results from above. Moreover, we employed a gene-centric approach, utilising all predicted and annotated genes for our analysis. The gene-centric approach typically provides a more comprehensive picture, as all genes account for a higher fraction of total reads than MAGs and their associated genes alone.

### Taxonomic assessment of metatranscriptomic samples

The metatranscriptomes were not depleted for rRNA, which allows taxonomic assessment based on the rRNA. The quality-controlled reads (from ATLAS, see above) were mapped to the SILVA rRNA database v138 [61] using Bowtie2 v2.3.4.1 [62] to quantify the abundance of different taxa within the soil communities.

### Metatranscriptomic analysis and quantitative metatranscriptomic

 Metatranscriptomes were quality-controlled using the ATLAS pipeline, and high-quality reads were mapped to all detected genes from the metagenomes using Bowtie2 v2.3.4.1 [62]. The functional annotation with eggNOG also annotates KEGG orthology IDs (KOs). The selection of metabolic marker genes related to the utilisation of both organic and inorganic electron donors, aerobic and anaerobic electron acceptors, oxygenic photosynthesis and light harvesting, carbon fixation, and nitrogen fixation was based on the reference gene databases from Chen et al. [38] and Bay et al. [41], and we kept the same colour nomenclature. In addition, genes involved in anoxygenic photosynthesis, specifically *pufL* and *pufM*, were included [32]. Detailed gene identifiers were obtained from the KEGG database (https://www.genome.jp/kegg/) and are summarised in Table S10.

 Normalisation of total counts of the mapped genes was performed using the Quantitative Metatranscriptomic (QT) method [63]. Each sample was normalised based on its total RNA content (µg g⁻¹ soil dry weight (dw); Table S1) using the known molecular weight of single-stranded RNA (3.40 × 10⁸ µg mol⁻¹ per 1,000 nucleotides) and Avogadro’s constant (6.02 × 10²³). Following Söllinger et al. [63], we assumed that mRNA constitutes 4% of total RNA. The total gene-mapped counts or specific gene counts were then adjusted by multiplying by the QT factor (transcript g^− 1^ soil; Table S9) for each sample.

### Phylogenetic analysis

Firstly, the Pfam motif PF00374 was identified in all genes associated with KEGG number K06281 on the Intrapro website: https://www.ebi.ac.uk/interpro/. Reference sequences of groups 1a, 1b, 1c, 1d, 1e, 1f, 1 g, 1 h, 1i, 1 L, and 2a were extracted from Bay et al. [41] and from http://services.birc.au.dk/hyddb/ [64]. For phylogenetic analysis, the amino acid sequences of genes associated with the KEGG number K06281 (encoding the large subunit of NiFe-type hydrogenase) were first aligned to reference sequences using ClustalW with default parameters. A maximum-likelihood phylogenetic tree was constructed using default parameters and bootstrapped with 10 replicates to visualise the evolutionary relationships among the sequences in MEGA11 [65].

### Data preparation, plots and statistical analyses

All community analyses and their relationship with environmental parameters were assessed in R software v4.0.5 [66]. For the analysis of the whole community (including prokaryotes and eukaryotes), samples were rarefied using the rtk package v0.2.6.1 [67] with a depth of 100 K reads. For the separate analysis of the prokaryotic and eukaryotic communities, samples were rarefied to depths of 100 K and 10 K reads, respectively. The rarefied data were converted to relative abundances with the R microeco package v1.2.0 using the *cal_abund()* function [68]. Hierarchical stacked barplots for bacterial community composition were generated using the microeco package and the *trans_abund()* function [68].

Non-metric Multi-dimensional Scaling (NMDS) based on Bray-Curtis dissimilarities was applied to explore beta diversity of the microbial community using the vegan package v2.6.4 [69] and visualised with the ggplot2 package v3.4.4 [70]. A PERMANOVA (Permutational multivariate analysis of variance) was implemented using the *adonis2*() function in the vegan package to identify significance in the different soil pits and depths. Additionally, depth and distance were used as continuous variables to assess and visually show how they influence the overall variation in the data. Significant parameters (*p* < 0.001) were identified using the *envfit*() function from the vegan package with 999 permutations, and only these parameters were included in the plots.

Additionally, the associations (with at least one significance of *p* < 0.001) between the microbial community and the metadata (subset from [54]; Table S1) were assessed using Spearman’s rank correlation. Correlations were calculated using the *cal_cor()* function of the microeco package, considering only taxa with relative abundance greater than 1.0%. Moreover, to visualise the transcriptomic profile and associated taxa, samples were transformed into a relative natural logarithm (LN) scale, and triplicates were merged for simplified visualisation. Heatmaps were made using the RColorBrewer v1.1.3 and plots v3.2.0 packages, and dendrograms were built based on the hierarchical clustering of taxa in the correlation matrix.

### Co-occurrence networks analysis

 The co-occurrence network analysis was performed using the taxa listed in Table S4, which were classified using the SILVA database (see Methods 2.4), as the taxonomic resolution of functional genes from the gene-centric approach was generally low and often limited to the phylum level. Co-occurrence networks were built based on Spearman correlation using taxa with a summed relative abundance (mean of triplicates) of > 0.05% across all samples and a correlation coefficient > 0.75, resulting in an undirected network with a scale‐free topology. Network construction and visualisation were carried out with R using the packages *igraph* v1.5.1 [71] and *RColorBrewer* v1.1.3 [72]. Network modules were detected using a random walk (function cluster_walktrap, *igraph* package), and the layout was calculated using the Fruchterman–Reingold algorithm (*layout.fruchterman.reingold()* function, *igraph* package) Pons and Latapy, 2005 [73]. Pairwise correlations among taxa were calculated using the *rcorr()* function within the *Hmisc* package v5.1.3 [74], and a Spearman correlation coefficient of R-value > 0.75 (*p* < 0.01) between two taxa was considered statistically robust. To assess the importance of individual nodes and modules within the network, two centrality measures were calculated: Degree Centrality and Betweenness Centrality [52]. Degree Centrality quantifies the number of direct connections (edges) a node has, reflecting its connectivity within the network. Betweenness Centrality measures the extent to which a node lies on the shortest paths between other pairs of nodes, highlighting its role as a critical intermediary in facilitating communication or information flow across the network [52]. Both centrality measures were computed using the *degree()* and betweenness() functions from the igraph package. Moreover, connections among different taxa were identified using the function *neighbors().* Nodes were classified as either prokaryotic or eukaryotic taxa based on their taxonomic assignment using the *ifelse()* function from the igraph package. Additionally, specific taxa were classified according to their functional roles as gas trace oxidisers (TGOs), oxygenic and anoxygenic photosynthesisers, or diazotrophs, based on reference taxa (cultivated and uncultivated taxa) known to possess the respective functional genes identified by Bay et al. [40], Imhoff et al. [32], and Ramond et al. [75]. For H_2_ oxidisers, only taxa known to contain the 1 h or 1 L group NiFe hydrogenases were considered, as these have been identified as the most relevant hydrogenase groups in desert environments [40–42]. These functional groups (Table S15) were extracted and visualised using the *subgraph()* function from the igraph package.

## Results

### RNA recovery, sequencing stats and β-diversity along the moisture gradient

RNA recovery was successfully achieved in these high-altitude mineral soils, with high recovery observed in the surface samples (Table S1). On average, RNA recovery was five times greater in the surface samples (0–5 cm) compared to the subsurface samples (20–30 cm; Table S1). Notably, RNA recovery in P3-1 was 5 and 10 times higher than in P1-1 and P5-1, respectively, while P4-1 showed recovery rates 4 and 9 times higher than P1-1 and P5-1 (Table S1). These RNA recovery patterns matched those observed for bacterial abundance (16 S rRNA gene copy number) and microbial activity (ATP content and fluorescein diacetate hydrolytic activities) in our previous research [54](Table S1), supporting our conclusions. This suggests that P3-1 and P4-1, located 8–23 m from the temporary lake along the moisture transect, represent the most metabolically active sites within the moisture gradient.

 Using samples from transect one (T1) as a reference, we generated eight metagenomic datasets, averaging 75,611,852 raw reads. After quality filtering, 0.04% of reads were assigned to known taxa in the SILVA database, and 55.37% mapped to predicted genes (Table S2). The metagenomic assemblies contained on average 134,928 contigs, totalling 384,029,042 bases, with an N50 value of 18,474.5, indicating moderate assembly contiguity (Table S3). Additional assembly statistics are provided in Table S3. On the other hand, we produced 24 metatranscriptomic datasets along the three transects, with an average of 93,597,694 raw reads. After quality filtering, 33.67% of reads were assigned to taxa in the SILVA database and 14.77% mapped to predicted genes (Table S2).

**Fig. 1:**
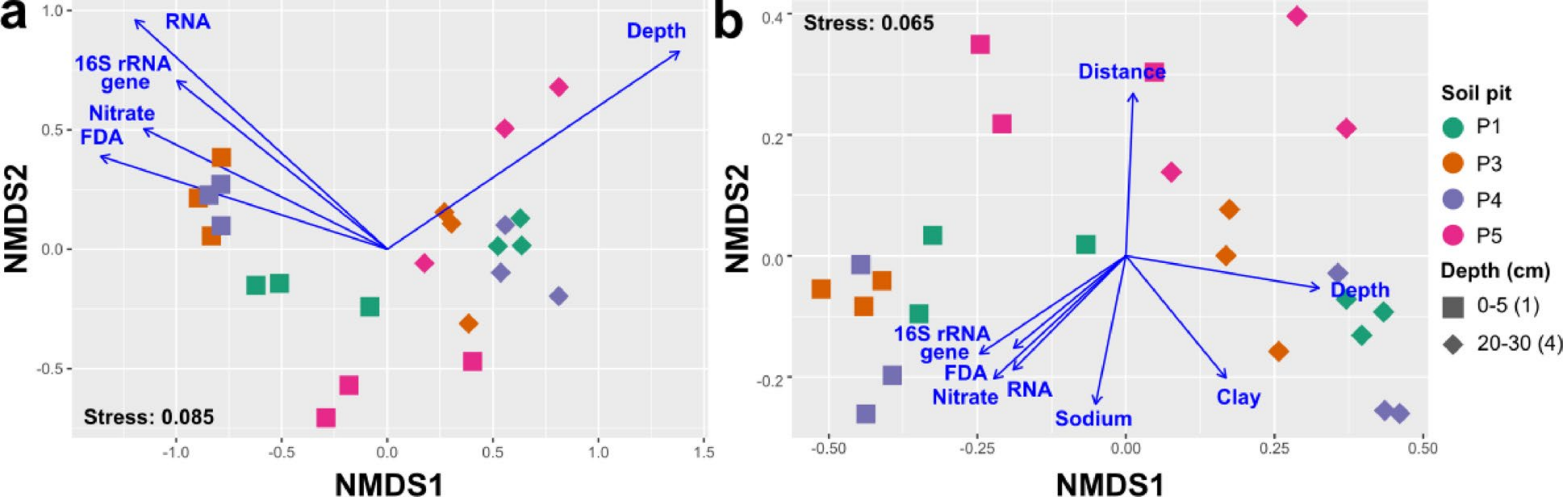
β-diversity of the microbial community along the moisture gradient in the Barrancas Blancas plain. Nonmetric multidimensional scaling (nMDS) was performed using Bray-Curtis distance with only significant parameters (*p* < 0.001). In panel (**a**), nMDS corresponds to the data mapped to the SILVA database, while in panel (**b**), it corresponds to the transcribed genes. Stress values are presented in the lower or upper-left corner. Colours indicate the different sites, and different shapes represent depth. Each point symbolises a biological replicate

**Fig. 2:**
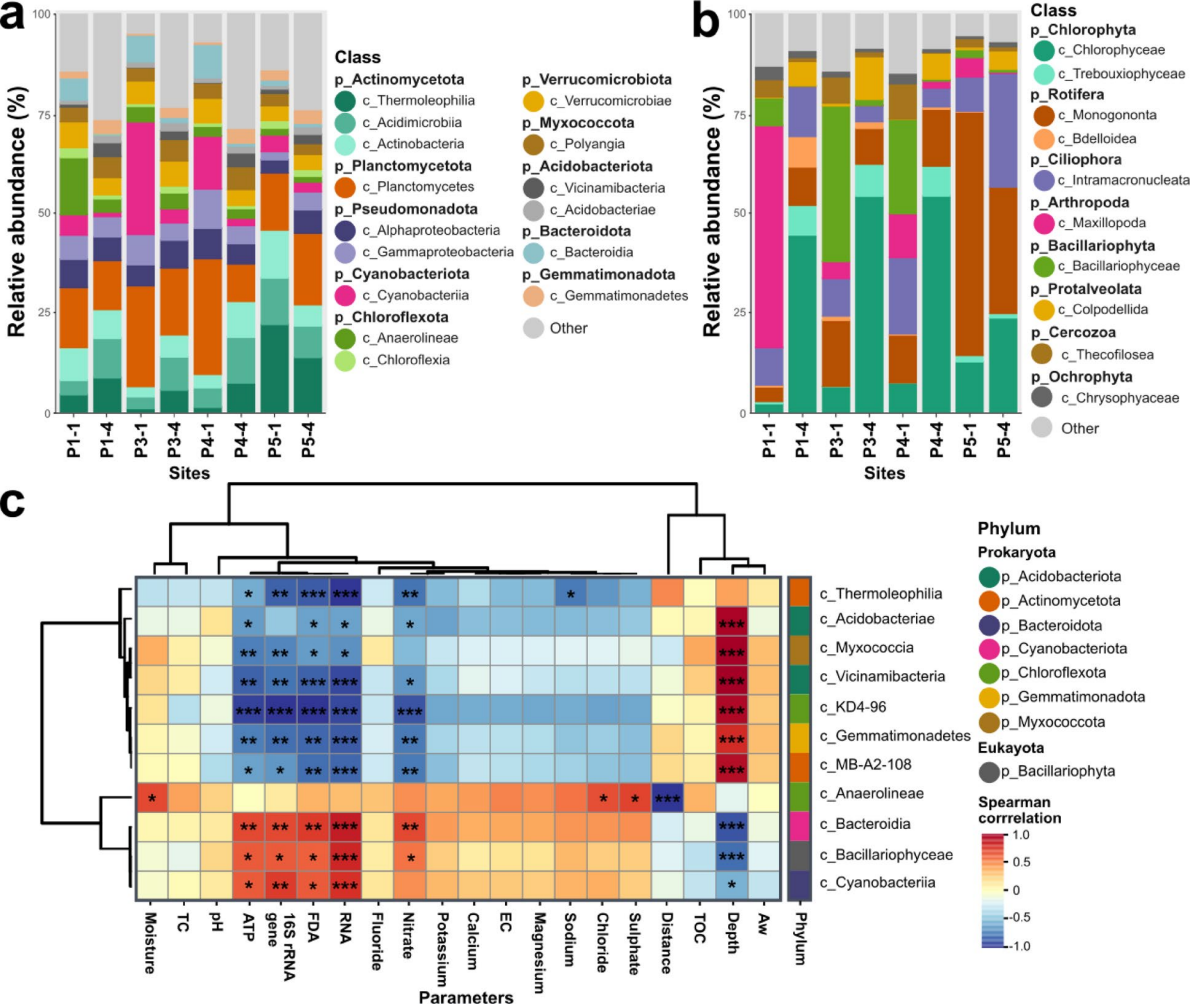
Microbial taxonomy of the transcriptomic data along the moisture transect. **a** Prokaryotic microbial abundance of the most abundant phyla (p) and their respective classes (c, in different colours) along the moisture transect. **b** Eukaryotic microbial abundance of the most abundant phyla and their respective classes (in different colours) along the moisture transect. **c** Heatmap of Spearman correlations between whole microbial community (Prokaryota and Eukaryota) at class level (phylum in different colours) and physicochemical parameters, microbial abundance, enzymatic activity, and RNA content. Significance is shown with asterisks (*p* < 0.05 =*; *p* < 0.01 = ** and *p* < 0.001 = ***), and rho’s Spearman correlation value is shown in brackets. EC: Electric conductivity; TC: total carbon; TOC: total organic carbon; AW: Water activity; and FDA: Fluorescein diacetate hydrolytic activity

 Non-metric multidimensional scaling (NMDS) was employed to evaluate β-diversity across soil pits along the moisture gradient (Fig. 1). When utilising data mapped to the SILVA database (Table S4), the location of soil pits was not significant (R^2^ = 0.1769, *p* > 0.05; Table S6), whereas depth exhibited a robust and statistically significant separation (R^2^ = 0.2540, *p* < 0.001; Table S6 and Fig. 1a). Conversely, NMDS analysis based on all transcribed genes (Table S5) illustrated a distinct separation of the reference site (P5) (R^2^ = 0.2414, *p* < 0.05; Table S6), along with a similarly strong clustering based on depth (R^2^ = 0.3273, *p* < 0.001; Table S6 and Fig. 1b). Regardless of the dataset employed, qPCR data, FDA, RNA content, and nitrate concentrations were found to correlate with the ordination axes, particularly clustering surface samples from P3 and P4 (Fig. 2a, b).

### Prokaryotic and eukaryotic taxonomy assignment along the moisture gradient

Metatranscriptomic analysis revealed that bacterial genes dominated transcription along the moisture transect, accounting for over 96% of total mapped gene counts per sample (Table 1). An increase in eukaryotic gene transcription was observed in surface samples, corresponding with the most active sites along the moisture gradient (Table 1). Comparatively, the reference site (P5) exhibited lower eukaryotic gene assignments than P1-1 (10 times lower), P3-1 (26.9 times lower), and P4-1 (16.8 times lower; Table 1). Archaeal assignment remained low, averaging 0.37% along the transect, with a slight inclination to increase with depth (Table 1). Additionally, non-assigned (NA) genes were minimal, averaging 0.15% across all samples (Table 1).

**Table 1:** Distribution of domains (in percentages) of Bacteria, Eukaryota, Archaea, and non-assigned (NA) along the moisture gradient

Sample ID	Soil pit	Depth (cm)	Bacteria (%)	Eukaryota (%)	Archaea (%)	NA (%)
P1-1	P1	0–5	98.49 ± 0.58	1.01 ± 0.55	0.29 ± 0.04	0.2 ± 0.06
P1-4	P1	20–30	99.09 ± 0.03	0.21 ± 0.03	0.51 ± 0.01	0.18 ± 0.01
P3-1	P3	0–5	96.79 ± 0.68	2.69 ± 0.69	0.21 ± 0.05	0.31 ± 0.04
P3-4	P3	20–30	99.17 ± 0.08	0.24 ± 0.04	0.46 ± 0.03	0.14 ± 0.01
P4-1	P4	0–5	97.71 ± 0.14	1.68 ± 0.22	0.15 ± 0.03	0.46 ± 0.11
P4-4	P4	20–30	99.08 ± 0.13	0.19 ± 0.03	0.5 ± 0.08	0.23 ± 0.03
P5-1	P5	0–5	99.55 ± 0.1	0.10 ± 0.02	0.31 ± 0.11	0.03 ± 0.01
P5-4	P5	20–30	99.15 ± 0.09	0.21 ± 0.07	0.53 ± 0.05	0.11 ± 0.02

The functional taxonomy of the microbial community, based on metatranscriptomes mapped to the SILVA rRNA database (see Methods 2.4), is presented in Fig. 2. The most abundant prokaryotic phyla included Actinomycetota, Planctomycetota, Pseudomonadota, Cyanobacteriota, Chloroflexota, Verrucomicrobiota, Myxococcota, Acidobacteriota, Bacteroidota, and Gemmatimonadota (Fig. 2a and Table S7). Among eukaryotic taxa, the dominant phyla were Chlorophyta, Rotifera, Ciliophora, Arthropoda, Bacillariophyta (diatoms), Protalveolata, Cercozoa, and Ochrophyta (Fig. 2b and Table S8).

The most notable finding in the prokaryotic dataset was the substantial increase in the abundance of the photosynthetic class Cyanobacteriia in the surface samples of the most active sites, which showed 28.4% and 13.5% relative abundance in P3-1 and P4-1, respectively. This represents a five- and two-fold increase compared to P1-1 and a six- and three-fold increase compared to P5-1 (Fig. 2a and Table S7). Interestingly, this contrasts with our previous findings [54], which reported only minimal cyanobacterial abundance. However, in P3-1, all three biological replicates consistently demonstrated a significant increase in cyanobacterial abundance (> 18% in all; Table S7), reinforcing the validity of this observation. Moreover, the class Bacteroidia showed consistently higher abundance on the surface, particularly in P1, P3, and P4 (Fig. 2a). Actinomycetota, represented by the classes Thermoleophilia, Acidimicrobiia, and Actinomycetota, exhibited a significant increase at the reference site P5 (Fig. 2a).

In the eukaryotic dataset, the class Maxillopoda (phylum Arthropoda) was notably the most abundant in P1-1, consistent with the presence of free-living aquatic crustaceans in the lake at the time of sampling, as described in [54]. More strikingly, the class Bacillariophyceae (phylum Bacillariophyta) showed a significant increase in abundance at the surface of the most active sites, P3 (39.42%) and P4 (23.79%), representing a five-fold and a three-fold increase compared to P1-1 and a 20-fold and a 12-fold increase compared to the reference site (P5-1; Fig. 2b and Table S8). On the other hand, the most relevant phyla in the subsurface were the green algae classes Chlorophyceae (phylum Chlorophyta), which exhibited more than 50% abundance in P3-4 and P4-4 (Fig. 2b). Moreover, the class Monogononta (phylum Rotifera) showed a substantial increase in P5 compared to the other sites (Fig. 2b).

In accordance with the observed trends, the increase in oxygenic phototrophs (Cyanobacteriia and Bacillariophyceae) demonstrated a positive correlation with the 16 S rRNA gene copy number, ATP content, fluorescein diacetate (FDA), and RNA content (Fig. 2c). Furthermore, the heterotrophic class Bacteroidia also exhibited a positive correlation with these parameters (Fig. 2c). Notably, both Bacillariophyceae and Bacteroidia displayed a significant correlation with the increasing nitrate content (Fig. 2c). Although no significant correlations were found with anoxygenic proteobacterial phototrophs, a slight increase was noted, primarily from the Alphaproteobacteria class (including orders Rhodobacterales, Rhodospirillales, Rhizobiales, and Sphingomonadaceae) within the surface samples of P3 and P4 (refer to Table S7). This last information is relevant for understanding the expression patterns in the subsequent section.

### Energetic strategies in the Barrancas Blancas plain

**Fig. 3:**
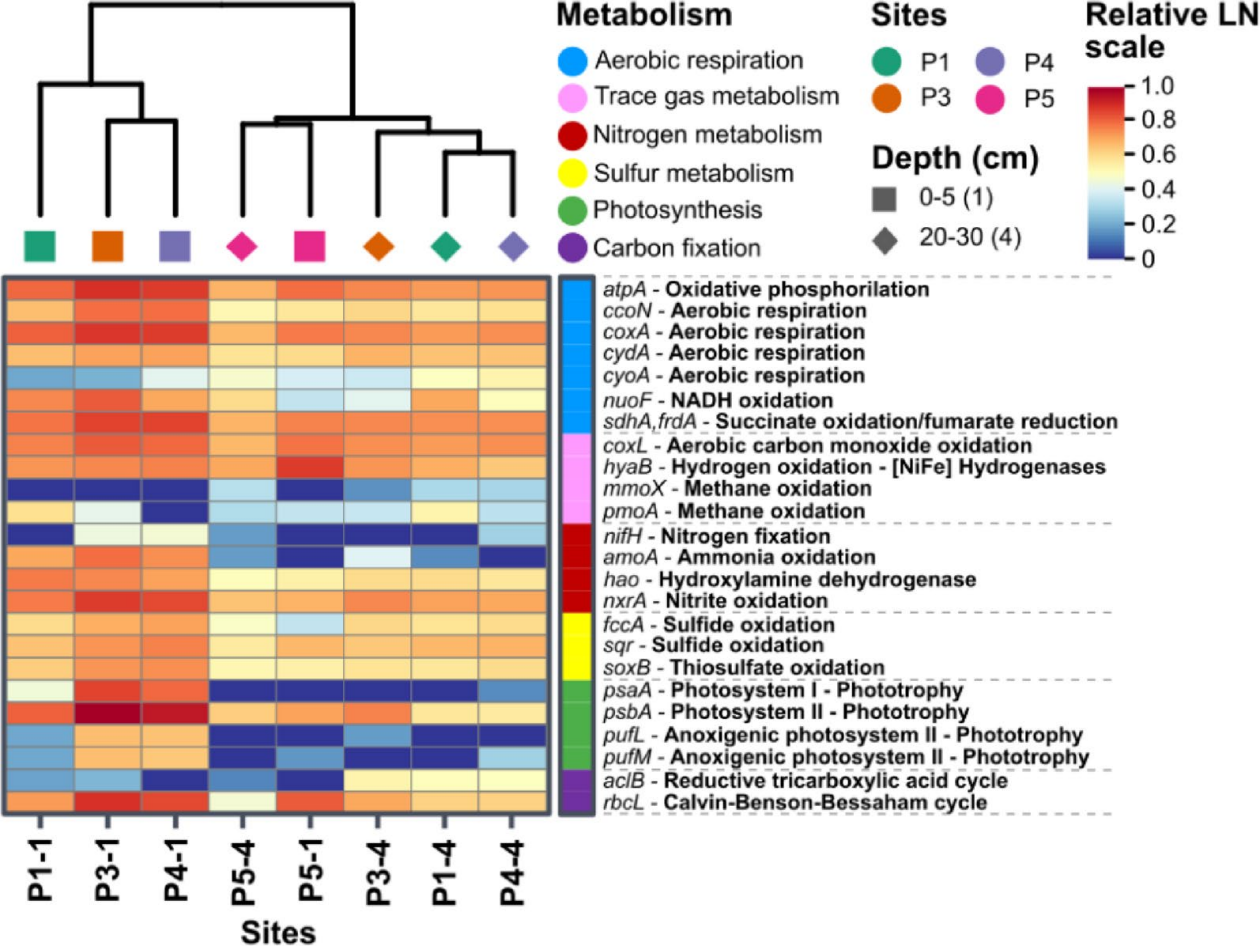
Heatmap of energy metabolism and other processes along the moisture transect in the BB plain. Different energy metabolisms are represented in different colours (top-right corner). Each process and its respective genes are described on the right side of the figure. Data was LN-transformed for better visualisation and comparison of data. Sites are shown in different colours, and depth is represented by different shapes. A dendrogram was created based on the hierarchical clustering in the correlation matrix

 Given the overall high RNA recovery and metabolic activity across the plain, particularly in the surface samples of P3 and P4, we investigated the key metabolic processes that enable the microbial community to thrive under the extreme environmental conditions of the BB plain (Fig. 3). For this purpose, gene count data were normalised using the quantitative metatranscriptomic (QT) method (Table S9) (see Methods, Sect. 2.5). The analysed genes, along with their corresponding KEGG identification numbers, are listed in Table S10.

Overall, the microbial community displayed high expression of key genes encoding terminal oxidases (*ccoN*, *coxA*, *cydA*, and *cyoA*; Fig. 3), underscoring its predominantly organoheterotrophic and aerobic characteristics. Additionally, transcripts related to the oxidation of both organic and inorganic energy sources were identified, including genes for sulfide (*fccA*, *fccB*), thiosulfate (*soxB*), ammonia (*amoA*), hydroxylamine (*hao*), and nitrite (*nxrA*) oxidation (Fig. 3). Furthermore, biological nitrogen fixation was indicated by the presence of the *nifH* gene (Fig. 3).

Notably, the community also expressed genes involved in the oxidation of atmospheric trace gases, including hydrogen (H_2_; *hyaB* gene–encoding the large subunit of [NiFe]-hydrogenases), carbon monoxide (CO; *coxL* gene), and methane (*mmoX* and *pmoA* genes; Fig. 3). However, [FeFe]-type hydrogenases were not detected in all samples (Table S10). Additionally, genes related to oxygenic photosynthesis, including those for photosystem I (*psaA*) and photosystem II (*psbA*), were present (Fig. 3). Although genes for anoxygenic photosystem I (*pscA*) were absent, genes encoding anoxygenic photosystem II (*pufL* and *pufM*) were detected (Fig. 3). Furthermore, key genes involved in various carbon fixation pathways were also assessed; however, only the genes for the reductive tricarboxylic acid cycle (aclB gene) and the Calvin-Benson-Bassham cycle (RuBisCO - *rbcL* gene) were identified (Fig. 3; Table S10).

In more detail, the transcriptional profiles of energy-related genes revealed several intriguing and significant patterns. Generally, most of genes were highly expressed in P1, P3, and P4 surface samples, which clustered together. In contrast, the subsurface samples and the reference site P5 formed a separate cluster, exhibiting generally lower expression levels (Fig. 3). Among the genes involved in trace gas oxidation, the *hyaB* gene, responsible for H_2_ oxidation, was the most highly expressed across the transect, particularly in the surface samples of the reference site (P5-1), where it reached 7.06 × 10^7^ transcripts of *hyaB* g^− 1^ soil dw. This represented a 17-fold increase compared to other samples (Fig. 3; Table S10). In contrast, the *coxL* gene, involved in CO oxidation, showed a different pattern, exhibiting an increasing trend towards the middle of the transect but without significant changes (Fig. 3; Table S10).

Regarding nitrogen metabolism, several notable patterns emerged as nitrite content increased toward the most active sites (Table S1), peaking at 5.70 mg L^− 1^ in P3-1, following a similar trend as RNA content and other biological parameters (16 S rRNA gene, ATP, and FDA; Table S1). Following, the nitrogen fixation gene was exclusively expressed in the soil pits P3 and P4, with the highest expression in surface samples: P3-1 exhibited 6.33 × 10^5^ transcripts of *nifH* g-1 soil dw, and P4-1 had 1.06 × 10^6^ transcripts (Table S10). The *amoA* and *nxrA* genes exhibited similar trends, with the highest expression in P3-1: P3-1, showing 1.46 × 10^6^ transcript *amoA* g-1 soil dw and 7.03 × 10^7^ transcript *nxrA* g-1 soil dw (Table S10). The gene hao, associated with hydroxylamine oxidation (nitrification), reached the highest values in P1-1, with 1.68 × 10^7^ transcript *hao* g-1 soil dw (Table S10).

Regarding photosynthetic genes, both oxygenic and anoxygenic photosynthesis genes exhibited an almost exclusive presence in surface samples, with significantly higher levels in P3-1 and P4-1 (Fig. 3; Table S10). Oxygenic photosynthetic genes, *psaA* and *psbA*, exhibited the highest transcript levels in P3-1, with 5.26 × 10^7^ transcript *psaA* g-1 soil dw and 1.0 × 10^9^ transcript *psbA* g-1 soil dw (Table S10). Notably, *psbA* had the highest transcript levels among all analysed genes, suggesting the importance of oxygenic photosynthesis in the BB plain (Table S10). Anoxygenic photosynthetic genes, *pufL* and *pufM*, exhibited a similar pattern, although with lower transcription values than the oxygenic genes (Table S10).

 Carbon fixation, primarily through the Calvin-Benson-Bassham cycle (CBB), was most prominent in P3-1, which exhibited the highest transcriptional levels of the RuBisCo gene *rbcL*, with 1.0 × 10^8^ transcripts g-1 soil dw (Table S10). This was followed by P4-1 and P5-1, which had values an order of magnitude lower, 4.39 × 10^7^ and 2.34 × 10^7^ transcripts per g-1 soil dw, respectively (Table S10). In contrast, P1-1 exhibited values two orders of magnitude lower than P3-1, with only 3.96 × 10^6^ transcripts per g-1 soil dw (Table S10).

### Phylogenetic analysis of hydrogenases

 We constructed a phylogenetic tree using the amino acid sequences of genes identified as hydrogenases (KEGG ID: K06281; Table S12), along with reference sequences representing different groups of [NiFe]-hydrogenases (1a, 1b, 1c, 1d, 1e, 1f, 1 g, 1 h, 1i, 1 L, and 2a; Table S12). Our analysis revealed that most identified hydrogenase genes clustered within the 1 h and 1 L groups (Figure S3) and that these groups accounted almost exclusively for the expression pattern of the hydrogenases (Figure S4 and Table S13).

### Microbial taxa associated with strategies in the Barrancas Blancas plain

**Fig. 4:**
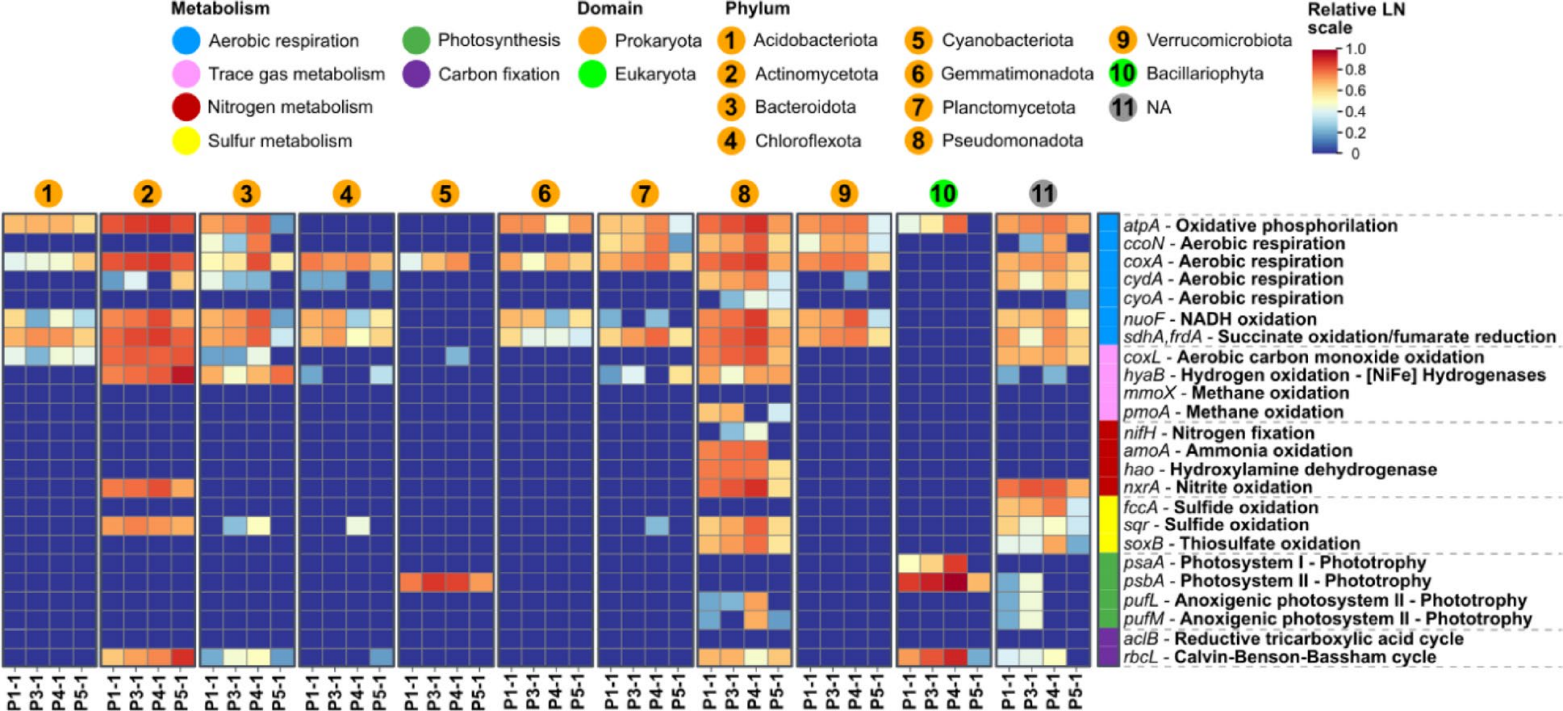
Heatmap of taxa associated with energy metabolism and other processes in the surface samples along the moisture transect in the BB plain. Nine of the most abundant prokaryotic phyla are highlighted and numbered from 1 to 9 (orange circles), while the eukaryotic microalga Bacillariophyta (diatoms) is represented as number 10 (green circle), and non-assigned (NA) taxa are shown as number 11 (grey circle). Different energy metabolisms are colour-coded (top-left corner). Each gene and its corresponding metabolic process are detailed on the right or top side of the figure, respectively. Data is presented on a relative natural logarithmic scale for better visualisation and to facilitate comparisons

 The taxonomic affiliation of energy-related genes (Table S11) is displayed in Fig. 4, highlighting the most abundant phyla on the surface. Regarding broader transcriptomic machinery, Pseudomonadota emerged as the most versatile phylum, capable of oxidising trace gases (CO and H_2_), sulfide, thiosulfate, ammonia, hydroxylamine, and nitrite (Fig. 4). Actinomycetota also exhibited a broad capacity to oxidise various compounds, followed by Bacteroidota, Chloroflexota, and Planctomycetota to a lesser extent (Fig. 4). Actinomycetota exhibited higher expression of the H_2_ oxidation gene (*hyaB*), followed by Bacteroidota, Pseudomonadota, and Chloroflexota. Regarding the expression profile, these phyla generally demonstrated increased transcription of the *hyaB* gene at the surface of the reference site (P5-1; Fig. 4).

Moreover, only Bacillariophyta displayed higher expression of both oxygenic photosynthesis-related genes (*psaA* and *psbA*), reaching peak expression in P3-1 and P4-1 (Fig. 4). Cyanobacteriota exhibited expression only of the key gene for oxygenic photosystem II (*psbA*; Fig. 4), indicating a lack of the entire photosynthetic system. On the other hand, anoxygenic photosynthesis genes were exclusively expressed by Pseudomonadota throughout the entire moisture transect, with a notably higher expression of *pufL* and *pufM* in P4-1 (Fig. 4).

 In terms of carbon fixation via the CBB cycle, Actinomycetota showed a significant increasing trend toward the reference site (P5-1), while Chloroflexota contributed only minimally, with expression detected solely in P5-1 (Fig. 4). Bacillariophyta exhibited a pronounced increase in P3-1 and P4-1, whereas Bacteroidota followed a similar trend but at lower levels (Fig. 4). Pseudomonadota maintained relatively consistent *rbcL* gene expression along the entire moisture transect. Interestingly, Cyanobacteriota did not express carbon fixation genes at the surface (Fig. 4) but did so in the subsurface (Figure S2).

 Genes related to trace gas oxidation and photosynthesis were also detected in the subsurface, albeit at lower expression levels (Table S11) and with a similar taxonomic affiliation as in the surface (Figure S2). Furthermore, non-assigned (NA) taxa exhibited the capacity to oxidise various compounds, including trace gases, and to fix carbon in both surface (Fig. 4) and subsurface samples (Figure S2). Photosynthetic genes in NA taxa were exclusively detected in surface samples (Fig. 4 and Figure S2), indicating an even broader photosynthetic community.

### Co-occurring patterns in the BB plain community

**Fig. 5:**
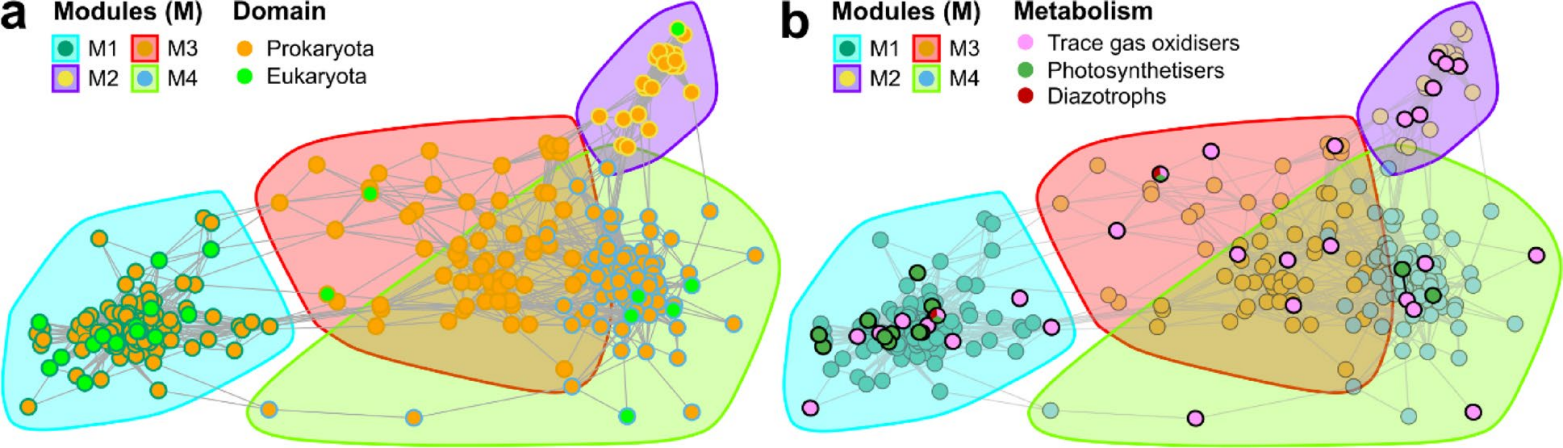
Co-occurrence network analysis of the functional microbial community in the BB plain. In (**a**) network indicating the presence of prokaryotic (in orange) and eukaryotic (in green) taxa. In (**b**), the presence of trace gas oxidisers (TGOs, in pink), photosynthetisers (in dark green), and diazotrophs (in red) is highlighted. As diazotrophic taxa also share TGO and photosynthetic properties, circles are divided into three colours. Each node represents a specific taxon, while the edges (lines) indicate connections between different taxa. Modules are colour-coded to distinguish different groups within the network

To identify potential interactions between prokaryotes and eukaryotes along the moisture transect, we constructed co-occurrence networks (Fig. 5a). Although experimental validation is required to confirm these relationships, identifying such potential interactions can help generate hypotheses and guide future research [37].

 The network was built using community members with an abundance greater than 0.05% while considering only significant correlations (*p* < 0.05, Spearman correlation). The resulting network comprised 287 nodes (taxa) and 3,805 edges (connections), with an average degree of 26.5 connections (node-edge ratio) and a moderately strong modularity score (Modularity = 0.57, Fig. 5 and Table S14). A total of four modules (M) were identified: M1 (104 members), M2 (21 members), M3 (63 members), and M4 (99 members; Table S15). M1 was the most eukaryote-dominated module, containing 14 ASVs representing 13.5% of the module population (green nodes in Fig. 5a, Table S15). In contrast, M2, M3, and M4 contained lower proportions of eukaryotic ASVs, accounting for 4.8% (1 ASV), 3.2% (2 ASVs), and 5.1% (5 ASVs), respectively (Fig. 5a, Table S15).

Based on abundance patterns and NMDS analysis (Figure S5), M1 was linked to the most active sites, with higher abundances found in P3-1 and P4-1 (Figure S6a). In contrast, M2 was mainly connected with the reference site (Figures S5 and S6b), while M3 and M4 were associated with deeper soil layers (Figures S5, S6c, and S6d). Accordingly, M1 showed a high presence of photosynthetic microorganisms, including Cyanobacteriota and Bacillariophyta (Figure S6a). Additionally, heterotrophic taxa were also abundant in this module, particularly the bacterial phyla Planctomycetota, Pseudomonadota, and Bacteroidota, along with the eukaryotic phyla Cercozoa, Ciliophora, and Rotifera (Figure S6a). As expected, M2 was predominantly made up of Actinomycetota and even showed the presence of the eukaryotic phylum Rotifera (Figure S6b). Meanwhile, M3 and M4 were primarily dominated by Actinomycetota, Myxococcota, Planctomycetota, Pseudomonadota, Chloroflexota, and the eukaryotic phylum Chlorophyta (Figure S6c and S6d). In general, the co-occurrence of photosynthesisers, heterotrophic prokaryotes, and heterotrophic eukaryotes reflects possible interconnections between these two domains (Fig. 5a). This was particularly interesting at the drier site, where eukaryotic taxa (Rotifers) co-occurred mainly with Actinomycetota, without the presence of photosynthesisers (Figure S6b).

 To further analyse the role of primary producers, we identified key taxa at the genus level, focusing on phototrophs, trace gas oxidisers (TGOs), and diazotrophic taxa. These identifications were based on previously characterised taxa known to possess the respective functional genes (Table S16). Additionally, we focused only on taxa that showed expression of *psaA*/*psbA* (for oxygenic photosynthesis) and *pulL*/*pufM* (for anoxygenic photosynthesis) for phototrophs, expression of H_2_ (*hyaB*), CO (*coxL*), or CH_4_ (*pmoA*) genes for TGOs, and expression of *nifH* for diazotrophic taxa in the section above (Fig. 4). In total, we identified 14 nodes as phototrophs, 28 as TGOs, and 2 as diazotrophs (Fig. 5b). Regarding phototrophs, they were found in all modules except M2 (the reference site) and had particularly high prevalence in M1 (10.6%; Fig. 5b). Conversely, TGOs were notably abundant in M2 (28.6%; Table S16), in agreement with the *hyaB* gene expression pattern described above (Fig. 4).

Interestingly, based on the identified groups, we found two Pseudomonadotal genera, *Bradyrhizobium* (Taxa_ 03445) and *Sphingomonas* (Taxa_03683), that have the potential to function as anoxygenic phototrophs, TGOs, and diazotrophs (nodes in green, pink, and red; Fig. 5b). From these results and the gene expression pattern noted above (Fig. 4), we could also suggest that photosynthesis and trace gas metabolism occurred simultaneously. Moreover, *Bradyrhizobium* (Taxa_ 03445) was directly connected to Taxa_03730 (Cyanobacteriota) and Taxa_04779 (Bacillariophyta), indicating a direct connection between phototrophs and diazotrophs (Table S16).

 In more detail, we analysed the degree of centrality on the selected nodes, which measures the number of direct connections (edges) a node has. Consequently, nodes with higher degree centrality are connected to more nodes, making them potentially important hubs [52]. Additionally, we calculated the betweenness centrality, which reflects how often a node appears on the shortest path between other nodes. High betweenness centrality indicates that the node is critical for network connectivity [52]. Phototrophs had an average degree of centrality of 31.9, compared to 19.8 for TGOs, highlighting the greater connectivity of phototrophs, particularly from Cyanobacteriota (Taxa_03730) and Bacillariophyta (Taxa_04779), which demonstrated 60 direct connections with other members (Table S16). However, the average degree of centrality for TGOs in M1 was 30.3, indicating their superior connectivity in the most active sites. Regarding betweenness, on average, TGOs had significantly higher values than phototrophs, suggesting their essential role in network connectivity (Table S16).

## Discussion

### Unprecedentedly high RNA recovery in the Puna de Atacama

The availability of liquid water is essential for sustaining microbial activity in high-altitude cryospheric environments [76–78]. Even though, the conditions in the Puna de Atacama, where the Barrancas Blancas (BB) plain is located, are characterised by overall strong negative water balance [3], the availability of liquid water through the presence of the temporary lake in the study area establish a unique environment which allowed high levels of microbial activity demonstrated in our previous research [54] which was validated by metatranscriptomic analysis of this study. This unique setting allowed us to successfully recover RNA from soil samples (Table S1). In particular, higher RNA concentrations were measured in the surfaces of P3 and P4 (Table S1), which aligns with our previous findings of increasing bacterial abundance and microbial activity in the same locations [54]. These results are particularly remarkable when compared to other poly-extreme environments, such as University Valley [79] and Beacon Valley in the McMurdo Dry Valleys (Antarctica) [80], or to the hyperarid Namib Desert (Namibia) [81], where RNA recovery from soil samples was either very low (even with pooled samples) or unsuccessful (even using up to 20 g of soil) [79]. In the lower altitude sites of the Atacama Desert, like the Yungay region in the hyperarid core of the desert, only intracellular DNA extraction was possible and used as a proxy to assess living and potentially active microorganisms, as overall low biomass led to extremely low RNA levels [19, 20], highlighting the difficulties of extracting RNA from desert environments. Our results indicate that the special conditions provided by the temporary lake led to an unusually high abundance of microbial activity, which allowed us to further explore the microbial transcriptomic profile, an approach that has never been done before in this area.

The higher surface RNA recovery was reflected in a strong depth-dependent community clustering rather than along the transect. Despite the well-defined moisture transect designed (from approximately 40% to approximately 10% water content; Table S1), the location (soil pit) had only a minor effect, which was only observed when analysing the transcribed gene dataset (*p* < 0.05; Table S6; Fig. 1b). In contrast, depth consistently showed a strong influence independently of the dataset (*p* < 0.01; Table S6; Fig. 1). In these high-altitude volcanic soils, despite large surface temperature fluctuations and other extreme surface conditions, the surface layer remains the most active, as subsurface microbial activity is limited by permanently subzero temperatures caused by the high insulation effect [5, 27]. This is likely one explanation for the higher RNA recovery at the surface and the strong depth effect observed in the b-diversity analysis. For this reason, Schmidt et al. [5] concluded that the growth and activity of the subsurface microbial community were completely inhibited, thereby explaining why the buried mummies found at the summit of Llullaillaco Volcano (6739 m a.s.l., Puna de Atacama) remained completely intact even without chemical preservation. Together with our results, these observations underscore that microbial activity in high-altitude Atacama soils is largely concentrated at the surface, highlighting its critical role in local biogeochemical processes. Furthermore, the previously noted distance effect suggests a functional metabolic transition along the moisture transect, which will be explored in the following sections.

### Photosynthetic communities thrive in the most active sites

The surface conditions, especially in the most active sites, created a “*window of opportunity*” not only for photosynthetic prokaryotic species but also for eukaryotic microalgae (Fig. 2a and b). The high abundance of Cyanobacteriota contrasted sharply with the minimal presence observed in earlier DNA-based analyses in the same study site [54]. The discrepancy in abundance is likely due to methodological differences, as taxa that appear in low abundance in DNA-based methods may actually be more functionally active, as evidenced by our RNA-based data and by other studies [82, 83]. These findings highlight the importance of RNA-based analyses to accurately assess the ecological roles and true function of microbial communities in arid environments. However, their use remains limited due to the low RNA content in oligotrophic environments, as mentioned above.

Moreover, the previously unstudied eukaryotic community exhibited a notable increase at the surface (Table 1), particularly from the microalgae belonging to the phylum Bacillariophyta (hereafter referred to as diatoms) at the most active sites (Fig. 2b). Although their presence is rare in many studies [84–86], diatoms have been shown to thrive in environments with lower organic content [87] and to serve as key primary producers in such settings [88–90]. In this sense, key genes involved in oxygenic photosynthesis (*psaA* and *psbA*, encoding the core components of photosystems I and II, respectively) followed the same pattern as the appearance of photosynthetic species (Fig. 3). However, only diatoms exhibited clear evidence of a functional photosynthetic system as the *psaA* gene was not expressed in Cyanobacteriota, indicating potential oxidative damage and/or photo-inhibition of their photosystem I due to intense solar and UV radiation [21, 38, 91] but not completely discarding their photosynthetic capacity as their activity might depend on circadian cycles [92, 93]. Furthermore, our results provided simultaneous evidence of anoxygenic photosynthesis at the most active sites, with genes associated with this process (*pufL* and *pufM*) being actively transcribed exclusively within proteobacterial taxa (Figs. 4 and 5). Taxa that possess this strategy (photoheterotrophs) have an adaptive advantage as they can harvest extra energy from light [22]. As anoxygenic photosynthesis can utilise alternative electron donors instead of water—such as sulfur compounds—this may explain the higher expression of genes involved in sulfur oxidation at the most active sites (Fig. 3). This pattern suggests a potential coupling between sulfur oxidation and anoxygenic photosynthesis, consistent with previous findings [94], and may have contributed to the elevated microbial activity observed at these locations. Altogether, this suggests the critical role of light-driven metabolism and the coexistence of diverse photosynthetic strategies within the same microhabitats in the BB plain.

Additionally, we suggest that the presence of photosynthetic microbes may have been facilitated by a microbial community with diazotrophic capacity, which was found in the most active sites (Figs. 4 and 5). Although total nitrogen content was below the detection limit across the entire moisture gradient [54], detectable nitrate values mirrored the *nifH* gene expression, which was restricted exclusively to the middle of the transect, as well as genes involved in nitrification (*amoA*, *hao*, and *nxrA*), which were expressed along the entire transect but showed higher expression in the mid-transect sites (Fig. 3). These patterns suggest that both nitrogen fixation and nitrification actively contributed to nitrogen availability at the most biologically active sites. While nitrate accumulation in the Atacama Desert has traditionally been attributed to atmospheric photochemical processes [95, 96], our findings suggest that, under the optimal conditions present at the most active sites, a community of diazotrophs and nitrifiers, primarily within the Pseudomonadota phylum, may also significantly contribute to nitrogen availability as reported in other cold desert ecosystems [97–99]. Furthermore, as nitrate content positively correlated with diatoms abundance (class Bacillariophyceae; Fig. 2c), we assume a potential link between nitrogen cycling and the development of the photosynthetic community. Nitrogen fixation is an essential process in nutrient-limited alpine environments, where it plays a key role in sustaining primary production [8, 75, 100], as its limitation is known to hinder phototrophic development [75, 101, 102]. Therefore, based on the observed capacity to mediate biological nitrogen fixation, we suggest that this may have contributed to or even fostered the significant development of the photosynthetically active diatom community at the most active sites in the BB plain.

These findings underscore the adaptive potential of surface photosynthetic microbes to develop, possibly as biological soil crusts (BSCs), under the optimal conditions of the most active sites. However, since we did not observe evident BSCs during the campaign, we suggest that these structures may be either cryptic, in an early developmental stage, or even disrupted by extreme environmental conditions, such as intense winds (up to 133 km h^− 1^) [103]. BSCs are a vital and well-documented feature in drylands [40–42, 104–106], combining photosynthetic and diazotrophic strategies that contribute to primary production [75, 107]. In this sense, and regardless of the undetectable organic carbon levels [54], we observed an increase in RuBisCO transcripts (*rbcL* gene) from the Calvin-Benson-Bassham (CBB) cycle at the most active sites, the primary pathway to fix CO_2_ into organic carbon in drylands [38–42, 94]. Moreover, our results indicated that photoautotrophic CO_2_ fixation in the BB plain primarily depends on diatoms, with anoxygenic phototrophs playing a complementary role. This finding contradicts the significant role in primary production that the latter have been attributed to in DNA-based studies in other Andean ecosystems [15, 31, 33, 34, 36, 37], emphasising the importance of exploring eukaryotic microbes.

These results provide significant new insights into the diverse range of photosynthetic strategies observed, from oxygenic and anoxygenic photosynthesis to nitrogen and carbon fixation. It highlights the role of both prokaryotic and eukaryotic organisms [108] in shaping high-altitude, arid biospheres under extreme environmental constraints.

### Trace gas metabolism drives microbial communities in the driest site

In contrast to the patterns observed at the most active sites, the driest location along the gradient shifted towards a transcriptional state associated with the oxidation of inorganic substrates, including sulfur and nitrogen compounds, and particularly hydrogen, as indicated by the high expression of group 1 h and 1 L nickel-iron (NiFe) hydrogenases (Fig. 3; Figure S4). Our results aligned with recent reports that indicated hydrogen (H_2_) oxidation as crucial process in dry environments, as it provides a stable energy source to sustain fundamental microbial metabolic functions [22, 40, 45]. Furthermore, the almost exclusive detection of groups 1 h and 1 L in the BB plain confirms their role as core functional groups in desert environments [40–42, 47], supporting the idea of shared survival strategies across poly-extreme environments.

The observed shift from a photosynthetic to a chemotrophic microbial community not only supports our initial hypothesis but also reveals a highly sensitive microbial response to moisture content over a remarkably short spatial scale (within meters). Similar responses have previously been reported under controlled experimental conditions [42] and across broader aridity gradients, such as the 160 km transect studied by Bay et al. [41]. Therefore, the distinct separation of the driest site from the wetter locations underscores the influence of local water dynamics along the BB plain.

Moreover, the transcription of hydrogenases was associated with a diverse bacterial community, primarily led by Actinomycetota (Fig. 4). The dominance of Actinomycetota at the driest site may be attributed to its remarkable ability and flexibility to oxidise atmospheric H₂ during extreme organic carbon starvation, as observed in other oligotrophic desert environments [38, 40–42, 44, 45, 109, 110]. Although the capacity for H₂ oxidation and utilisation of other inorganic substrates was also observed at the middle sites of the transect, the energy derived from them likely supported aerobic respiration [47], fuelled by organic compounds—such as exudates from the photosynthetic community [111, 112]— and partially carbon fixation through chemolithoautotrophy. Importantly, because H₂ can also be generated in situ through processes such as fermentation by heterotrophic taxa and nitrogen fixation by diazotrophs [113], its availability may be higher than expected, potentially making H₂ an even more ecologically relevant energy source in these high-altitude soils along the entire transect.

In this context, the detection of carbon fixation capacity at the reference site indicated the potential capacity of the microbial community to grow chemolithotrophically when water is scarcer, independently of the photosynthetic community. However, due to the energetic constraints of coupling atmospheric H₂ oxidation to CO₂ fixation, this process has been theoretically estimated to support only slow biomass formation [40, 41, 47, 114]. Together with recent findings by Garvin et al. [115], our finding indicates that the Puna de Atacama hosts a diverse and metabolically flexible community, with the capacity to thrive in drier locations through chemolithotrophy, particularly by utilising atmospheric trace gases.

### Community assembly and trophic interactions

**Fig. 6:**
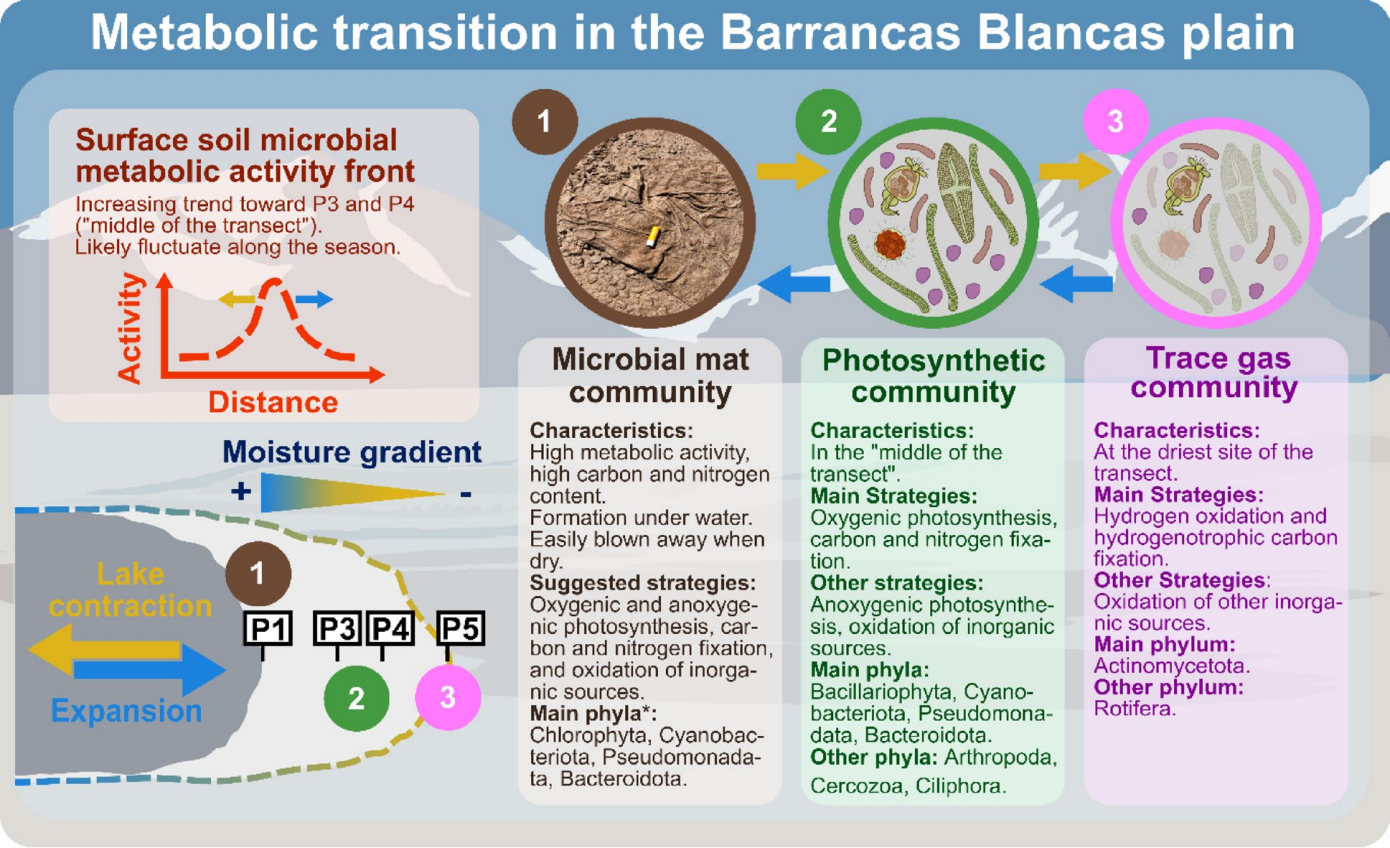
Conceptual scheme of metabolic transition and primary production dynamics in the Barrancas Blancas plain. This hypothetical scheme (not to scale) indicates that lake dynamics (in yellow and blue arrows) may determine the community’s metabolic state. Under drier conditions (reference site, P5), the trace gas community thrives (number 3 in pink). As the lake expands and water conditions are optimal (higher metabolic activity in the middle of the transect), the community might transition towards the development of the photosynthetic community (number 2 in green). Furthermore, the presence of photosynthetic microbial mats (number 1 in brown) along the lake suggests that the microbial community could possibly transition into these structures in response to lake dynamics, likely under water [128], though further research is needed to confirm this. Moreover, this assembly-disassembly dynamic likely occurs every season when the lake is present. The front of microbial metabolic activity (orange dashed line) reflects ecosystem adaptation to hydrological changes. *: Data based on 16 S and 18 S rRNA gene amplicon data [54]

Taking the previous result together, we propose that moisture variability along the gradient is a key ecological driver of microbial community assembly, selectively enriching functionally diverse microbial guilds and facilitating the formation of increasingly complex trophic networks under optimal moisture conditions (Fig. 6). Our previously stated hypothesis regarding the relationship between nitrogen cycling and the development of the photosynthetic community was validated through co-occurrence analysis. Our results revealed a direct connection between potential proteobacterial diazotrophic taxa (genus *Bradyrhizobium*) and diatoms (Table S16), fostering mutualistic relationships. The appearance of diazotrophic taxa may have been essential for photosynthetic community development, leading to greater species diversity as new niches become available [116–118]. A recent finding showed that the relationship between an alphaproteobacterium (Rhizobiales class) and a diatom is crucial for photosynthetic carbon fixation in the ocean [119]. In turn, the presence of Cyanobacteriota and diatoms may have promoted the establishment of other microbial members, acting as essential hubs (high connections to other taxa; Table S16) and enhancing community trophic complexity. For example, the association between Cyanobacteriota and Bacteroidota in marine consortia illustrates this dynamic, where Bacteroidota degrade complex extracellular polymers produced by Cyanobacteriota [22]. Additionally, ciliates (Ciliophora) and cercozoans (Cercozoa), abundant grazers in the most active sites, are common in freshwater systems [120, 121], indicating the presence of predator/prey relationships, as Cercozoans are bacterial predators and can even parasitise phototrophic microalgae [122, 123]. Moreover, calanoid copepods (*Eudiaptomus* genus) [54] also co-occurred in the photosynthetic module (M1; Figure S6a). These copepods feed on algae, ciliates, and even on bacteria [124], likely dominating the trophic levels in the BB plain. Our previous research suggested that under unfavourable conditions, such as lake contraction, they may enter the microbial loop, serving as a nutrient source for heterotrophic phyla like Bacteroidota and Pseudomonadota, which can degrade complex polymers and complete the trophic cycle [54].

Under the more “unfavourable” conditions at the surface of the reference site, the trace gas oxidiser community may support the community even across different trophic levels. Overall connectivity (degree and betweenness; Table S16) of trace gas oxidisers in the BB plain was much higher than in another high-altitude study [52], suggesting a greater impact on these microbes within the BB plain microbial community. Moreover, network analysis revealed that even eukaryotic members (Rotifera) co-occurred within the reference (M2, Figure S6b). Rotifers are known predators in microbial food webs, and feeding experiments suggest they can even consume bacteria [125]. Additionally, experimental data have shown that rotifers can be active even in high-altitude areas of the Puna de Atacama [126]. Although trace gas metabolism is generally considered to yield limited energy, the small carbon fixation rate (resulting in biomass production) reported in some studies [40, 47, 114] may still contribute nutrients to other organisms. In predatory trophic relationships, this could occur through direct bacterial consumption. The biomass produced via trace gas metabolism (possibly in combination with other inorganic sources) [127] may serve as a food source for different predators, and it is possible that an active chemolithotrophic community provides sufficient energy and resources to sustain rotifers at the reference site, although this hypothesis requires further investigation. Therefore, the recent discoveries on primary production by trace gas oxidisers [45] introduce a new paradigm in microbial ecology [52], raising questions about their role in supporting microbial communities and shaping trophic interactions.

Moreover, the BB plain also hosted highly productive photosynthetic microbial mats near the shorelines of the temporary lake, as reported previously [54]. These structures may accumulate carbon-rich polymers when photosynthesis exceeds consumption, forming a matrix that attracts a diverse array of microbial guilds [128]. We hypothesise that the microbial community present in the BB plain might be able to transition from a desertic community into a photosynthetic state (likely as BSCs) and, in combination with the lake’s expansion, contraction and underwater dynamics, possibly transition into microbial mats (Fig. 6). Recent work on microbialites—structurally analogous to microbial mats—demonstrates that molecular hydrogen functions as a key metabolic currency supporting diverse microbial guilds [113], suggesting that similar processes may also operate within mat structures at the BB plain. Moreover, when exposed to dry conditions, these mats can be easily carried by strong winds to remote places [26, 128–130]. Therefore, it is very likely that this assembly-disassembly dynamic occurs every season when the lake appears. Similar community assembly-disassembly has been observed by Lindemann et al. [128], though in semi-arid ecosystems. Our results further emphasise that the influence of microbial and trophic interactions may be more significant than traditionally assumed, potentially extending beyond the predominant focus on environmental selection, as also mentioned in other studies [37, 116, 131]. The interactions and dynamics of the lake during both expansion and contraction phases (Fig. 6), along with the underwater dynamics, necessitate deeper exploration. Future research should emphasise understanding the links between basic metabolism (such as trace gas metabolism) and trophic interactions.

## Conclusion

The Barrancas Blancas (BB) plain presents a unique natural laboratory for investigating how moisture availability shapes microbial metabolism and community structure in a poly-extreme environment. Along the moisture gradient imposed by a temporary lake, we observed a striking increase in transcriptomic activity at the most active sites (P3 and P4), particularly at the surface. These sites were dominated by a photosynthetic microalgal community (Bacillariophyta) and exhibited high expression of genes involved in oxygenic (*psaA*,* psbA*) and anoxygenic (*pufL*,* pufM*) photosynthesis, carbon fixation (*rbcL*), and nitrogen fixation (*nifH*) - highlighting the presence of active primary production despite extremely low levels of detectable organic carbon and nitrogen. In contrast, the surface of the drier site (P5), characterised by Actinomycetota adapted to arid conditions, showed a metabolic profile dominated by oxidation of inorganic sources, especially hydrogen oxidation (*hyaB*) and associated hydrogenotrophic carbon fixation. This shift in dominant energy and carbon acquisition strategies along the transect - from trace gas-based metabolism to light-driven primary production - illustrates a clear functional transition in microbial communities in response to moisture availability, even over a relatively short distance. The BB moisture gradient thus acts as a powerful ecological filter, driving community assembly by selectively enriching for microbial guilds with specialised metabolic capabilities. Under more favourable conditions, this leads to increased functional complexity and the emergence of more complex trophic interactions. Notably, even at the driest site, evidence of trophic structuring was found, likely based on trace gas metabolism, underscoring the ecological significance of energy-limited survival strategies in the desert biosphere. Taken together, our findings demonstrate that the BB plain is an exceptional model system for exploring how fundamental microbial metabolisms shape community structure and function across environmental gradients. This work provides new insights into the flexibility of primary production mechanisms in extreme environments and expands our understanding of how life persists and interacts at the very edge of habitability.

## Supplementary Information

Supplementary Material 1

Supplementary Material 2

Supplementary Material 3

## Data Availability

The data supporting the findings of this study are available in the European Nucleotide Archive (ENA) under project ID PRJEB89784 on the following link: [https://www.ebi.ac.uk/ena/browser/view/PRJEB89784](https:/www.ebi.ac.uk/ena/browser/view/PRJEB89784).

## Abbreviations

BB: Barrancas Blancas
CBB: Calvin-Benson-Bassham cycle
iDNA: Intracellular DNA
M: Module
NA: Non-assigned
P: Soil pit
RuBisCO: Ribulose-1,5-bisphosphate carboxylase/oxygenase
T: Transect
