# Supplementary material for: Metabolic transitions along a moisture gradient in a poly-extreme high-altitude desert ecosystem within the Atacama Desert
Source: Environ Microbiome. 2026 Jan 23;21:20. doi: 10.1186/s40793-025-00847-7 (PMC12849286; doi:10.1186/s40793-025-00847-7)
Supplement: Supplementary file 2 — Supplementary Material 2 [file 40793_2025_847_MOESM2_ESM.docx]

***Journal: Environmental Microbiome***

**Metabolic transitions along a moisture gradient in a poly-extreme high-altitude desert ecosystem within the Atacama Desert**

Diego Medina Caro^1^*, Alexander Bartholomäus^1^, Ayón García^2^, Rómulo Oses^3^, Susanne Liebner^1,4^ and Dirk Wagner^1,5^

^1^ GFZ Helmholtz Centre for Geosciences, Section Geomicrobiology, Telegrafenberg, 14473 Potsdam, Germany

^2^ Laboratorio de Investigación de la Criósfera de los Andes (LICA), Instituto de investigaciones científicas y tecnológicas (IDICTEC), Universidad de Atacama, 1530000 Copiapó, Chile

^3^ Centro Regional de Investigación y Desarrollo Sustentable de Atacama (CRIDESAT), Universidad de Atacama, 1530000 Copiapó, Chile

^4^ Institute of Biochemistry and Biology, University of Potsdam, 14476 Potsdam, Germany

^5^ Institute of Geosciences, University of Potsdam, 14476 Potsdam, Germany

**Supplementary figures**

***Figure S1:*** *Study site – Barrancas Blancas plain and temporary lake. a) Elevation map of the study site. b) Drone picture displaying the moisture gradient and samples selected for metatranscriptomic analysis. The legend on a) indicates the elevation, temporary lake, and uncovered glacier, while that on c) shows the colour of each transect and the depth of selected samples. T=transect, P=soil pit, LS=lake sediment.*


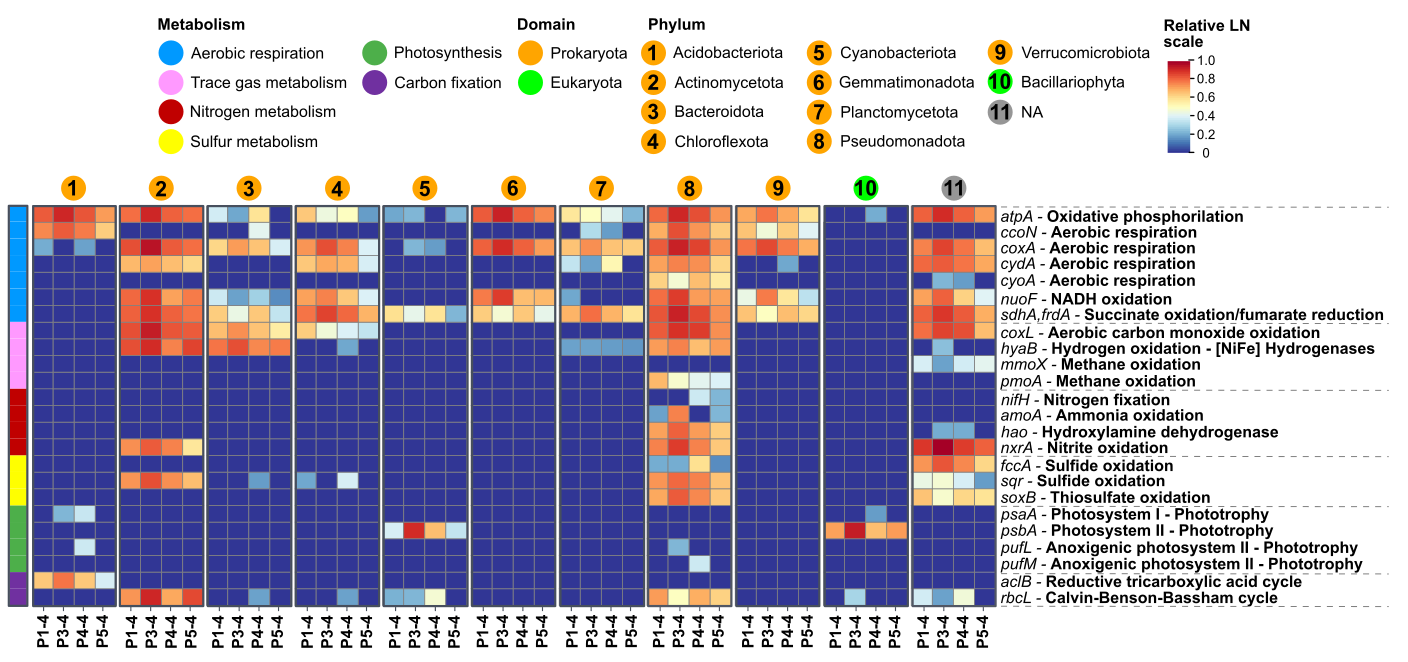


***Figure S2:*** *Heatmap of taxa associated with energy metabolism and other processes in the subsurface samples along the moisture transect in the BB plain. Nine of the most abundant prokaryotic phyla are highlighted and numbered from 1 to 9 (orange circles), while the eukaryotic microalga Bacillariophyta (diatoms) is represented as number 10 (green circle), and non-assigned (NA) taxa are shown as number 11 (grey circle). Different energy metabolisms are colour-coded (top-left corner). Each gene and its corresponding metabolic process are detailed on the right* *or top side of the figure, respectively. Data is presented on a relative natural logarithmic scale for better visualisation and to facilitate comparisons.*

**Figure S3 was uploaded as a separate file.**

***
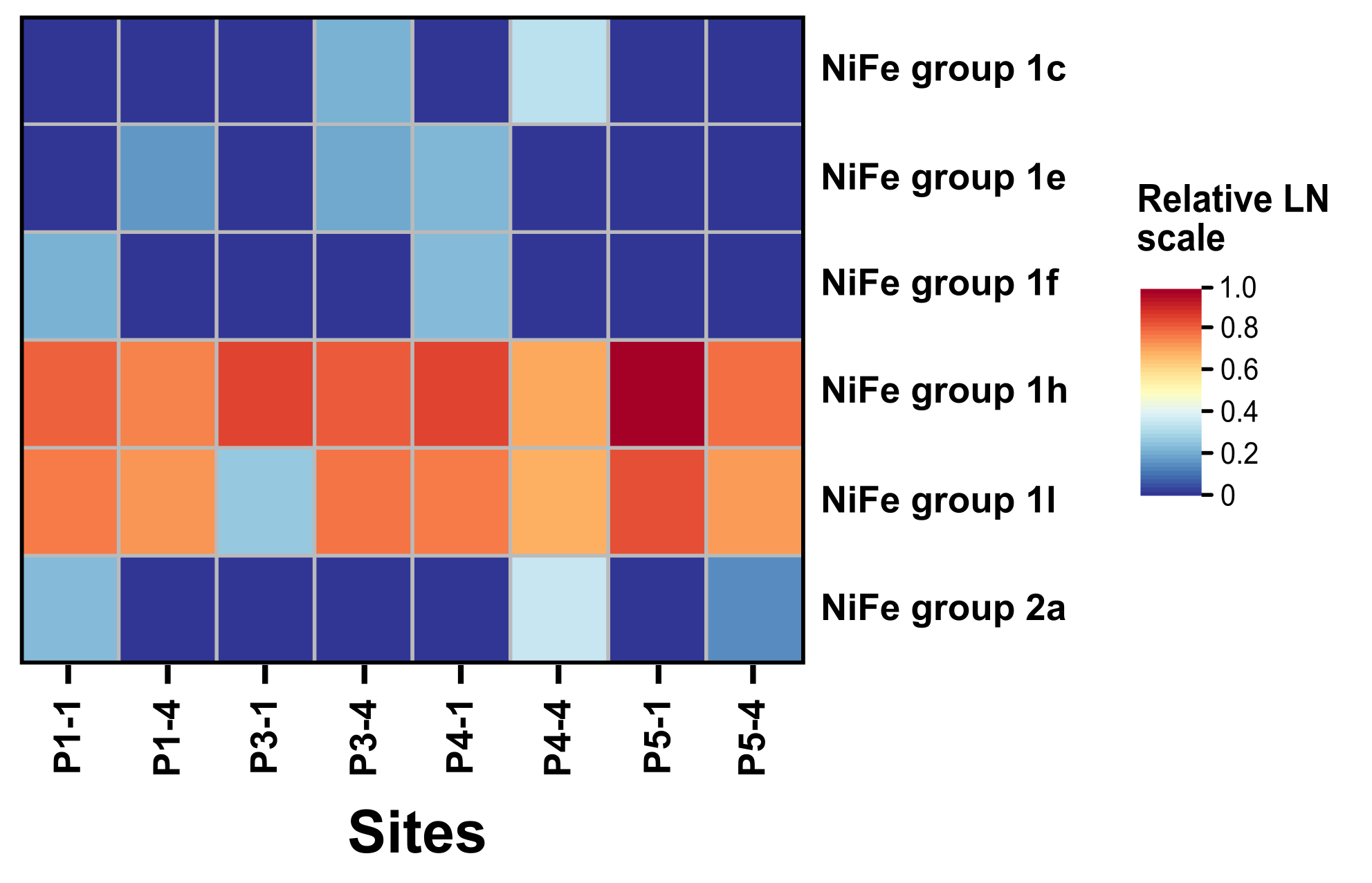
***

***Figure S4:*** *Heatmap of [NiFe]-hydrogenases groups along the moisture gradient in the Barrancas Blancas plain. Data is presented on a relative natural logarithmic scale for better visualisation and to facilitate comparisons.*


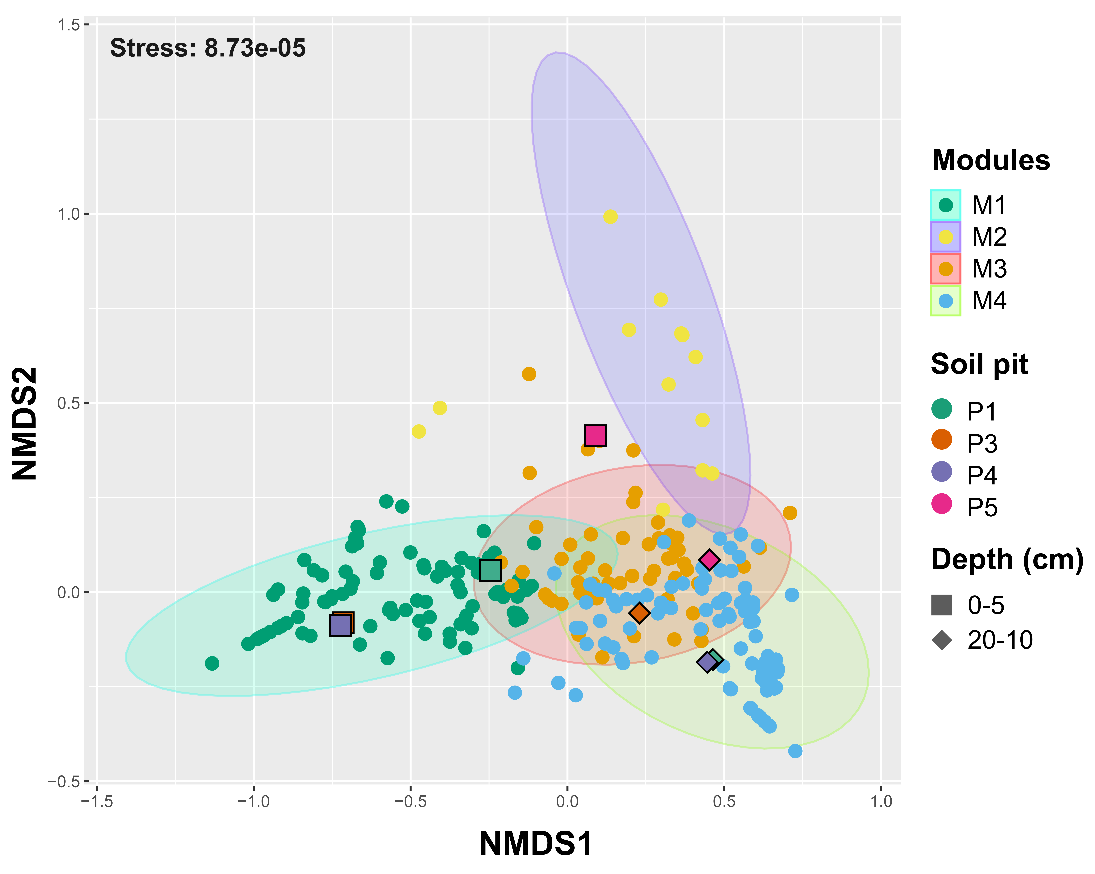


***Figure S5:*** *Nonmetric multidimensional scaling (NMDS)* *from network analysis. NMDS was performed using the 287 ASVs (circles) retrieved from the network analysis, based on the Bray-Curtis distance. Different colours represent modules and soil pits, while different shapes indicate depth. Stress is displayed in the upper left corner.* ***Note:*** *The stress value was low due to the limited number of samples and their high similarity. However, since the analysis was used solely to assign approximate locations to the different modules (along with Fig. S6), we have included it in the supplementary material for reference.*


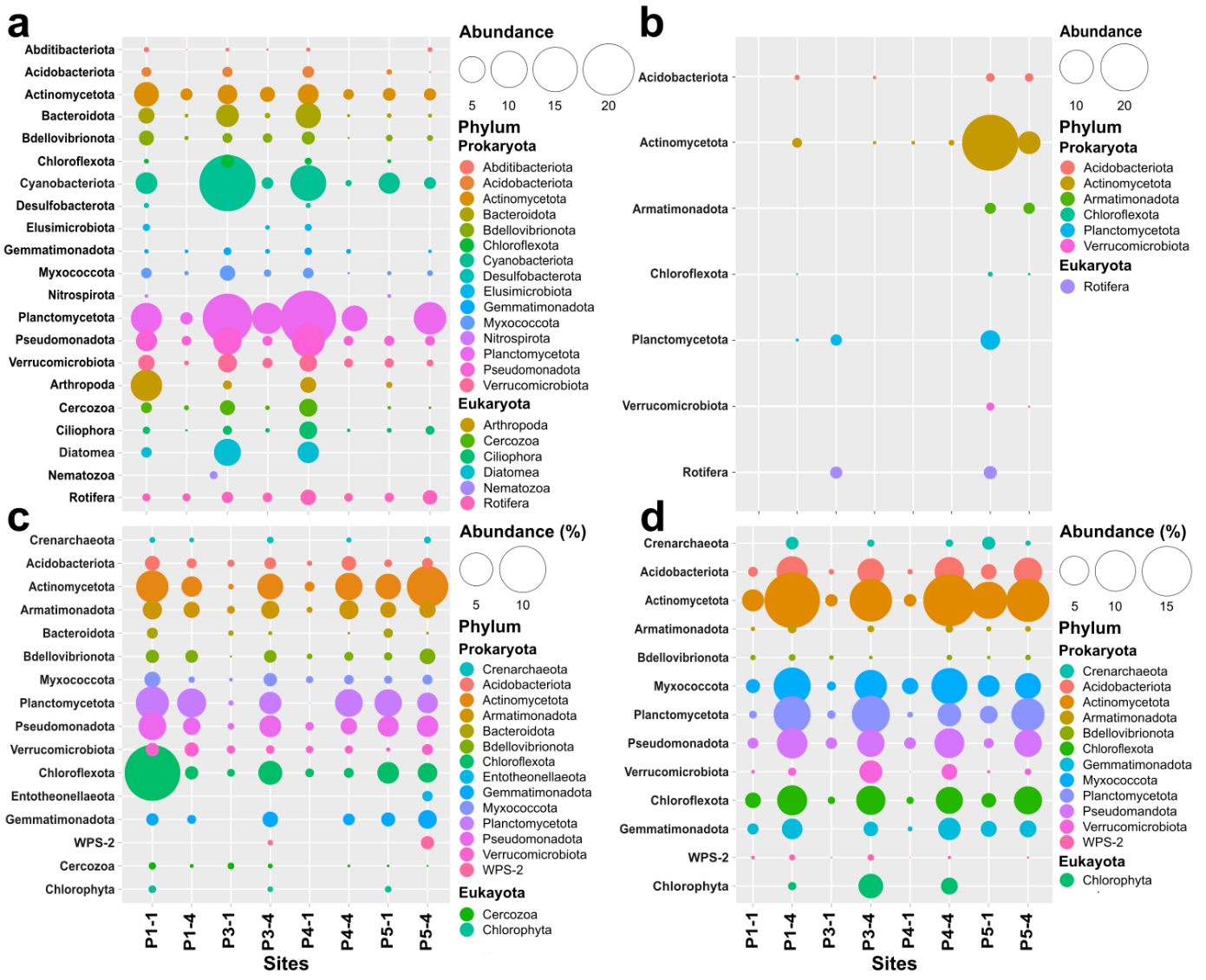


***Figure S6:*** *Bubble plot of the microbial composition of different modules of network analysis. Microbial composition at the phylum level across the moisture gradient and depth profiles of a) module 1 (M1), b) M2, c) M3, and d) M4.*
