# Supplementary material for: Metabolic transitions along a moisture gradient in a poly-extreme high-altitude desert ecosystem within the Atacama Desert
Source: Environ Microbiome. 2026 Jan 23;21:20. doi: 10.1186/s40793-025-00847-7 (PMC12849286; doi:10.1186/s40793-025-00847-7)

Figure S3: Phylogenetic identification of [NiFe]-hydrogenase groups in the Barrancas Blancas plain. A maximum-likelihood phylogenetic tree illustrates the sequence divergence of [NiFe]-hydrogenases associated with KEGG ortholog K06281, which encodes the large subunit of NiFe-type hydrogenases. Amino acid sequences retrieved from genes annotated as K06281 were aligned with reference sequences representing groups 1a, 1b, 1c, 1d, 1e, 1f, 1g, 1h, 1i, 1l, and 2a. Phylogenetic inference was performed using 10 bootstrap replicates. All sequences used in the analysis are listed in Table S10. The green line was used to divide the tree for better visualization.

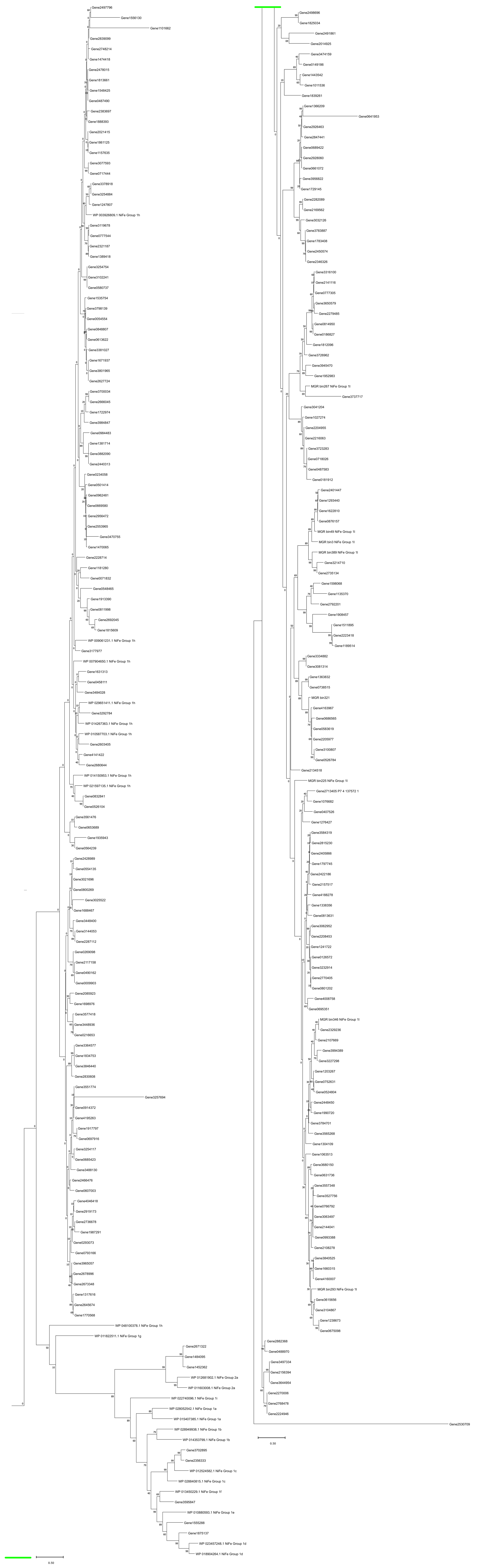

Supplement: Supplementary file 3 — Supplementary Material 3 [file 40793_2025_847_MOESM3_ESM.pdf]
